# High Temperatures Decrease the Flight Capacity of *Diaphorina citri* Kuwayama (Hemiptera: Liviidae)

**DOI:** 10.3390/insects12050394

**Published:** 2021-04-29

**Authors:** Carlos A. Antolinez, Tobias Moyneur, Xavier Martini, Monique J. Rivera

**Affiliations:** 1 Department of Entomology, University of California Riverside, 900 University Ave., Riverside, CA 92521, USA; carlosa@ucr.edu (C.A.A.); tobiasmoyneur@gmail.com (T.M.); 2 North Florida Research and Education Center, Department of Entomology and Nematology, University of Florida, Quincy, FL 32351, USA; xmartini@ufl.edu

**Keywords:** flight mill, citrus greening, HLB primary spread, heat tolerance, Asian citrus psyllid, vector dispersion, flight behavior

## Abstract

**Simple Summary:**

Flight capacity is directly associated with the spread of vector-transmitted plant pathogens. However, vector flight capacity could be affected by environmental conditions such as temperature and humidity. Here, we studied the effect of high temperatures and humidity in the flight capacity of *Diaphorina citri*, the main vector of the Huanglongbing disease of citrus. Our data suggest that high heat environments affect the propensity of *D. citri* to engage in long-distance flights, as well as the duration and distance flown, regardless of the humidity level. This information is fundamental to understand the dispersion of *D. citri* under different environmental conditions.

**Abstract:**

*Diaphorina citri* Kuwayama (Hemiptera: Liviidae), commonly known as Asian citrus psyllid (ACP), is an invasive insect pest and the vector of the bacterium causing Huanglongbing (HLB), a lethal disease of citrus. In the United States, ACP has been established in all citrus-producing zones, all of which have different environmental conditions. The spread of ACP and, more importantly, HLB, has progressed differently depending on the state, with more rapid spread in Florida and Texas than in California. Climatic variations between the regions are likely a strong factor in the difference in the rate of spread. Despite this, it is unknown how the flight capacity of *D. citri* is influenced by high temperatures (>30 °C) and subsequently, low humidity experienced in California but not in Texas or Florida. In this study, by using a custom-made, temperature-controlled flight mill arena, we assessed the effect of high temperatures on the flight capacity and flight propensity of *D. citri* under low (20–40%) and high (76–90%) relative humidity conditions. We found that temperature and humidity influence the propensity to engage in short or long-distance flight events. Psyllids exposed to temperatures above 43 °C only performed short flights (˂60 s), and a high relative humidity significantly decrease the proportion of long flights (≥60 s) at 26 and 40 °C. The flight capacity for insects who engaged in short and long flights was significantly affected by temperature but not by humidity. For long flyers, temperature (in the 26–43 °C range) was negatively correlated with distance flown and flight duration. The most favorable temperature for long dispersion was 26 °C, with suboptimal temperatures in the range of 32–37 °C and the least favorable temperatures at 40 and 43 °C. In conclusion, *D. citri* is able to fly in a broad range of temperatures and efficiently fly in high and low humidity. However, temperatures above 40 °C, similar to those experienced in semi-arid environments like Southern California or Arizona, are detrimental for its flight capacity.

## 1. Introduction

Flight capacity is a key component of determining the dispersal ability insects [1,2]. For pest management purposes, it is critical to identify the environmental conditions favoring or decreasing the dispersion of highly invasive insect pests. Ambient temperature and relative humidity can directly influence insect flight capacity [3,4,5,6]. However, depending on the insect species, flight activity is more efficient within a specific range of temperatures and humidity [1,7]. Accordingly, identifying the range of environmental conditions that permit the efficient flight activity of a given species is critical to understanding and thus predicting its dispersion.

The Asian citrus psyllid (ACP) *Diaphorina citri* Kuwayama (Hemiptera: Liviidae) is a pest native to tropical and subtropical Asia, but it has spread into the Americas and recently been reported in Africa [8,9]. In the United States, *D. citri* was first reported in Florida in 1998 [10]; subsequently, it has spread to the states of Arizona, Alabama, California, Georgia, Louisiana, Mississippi, South Carolina, and Texas [11,12,13,14]. *D. citri* is the vector of the of the lethal, phloem-restricted bacterium *Candidatus* Liberibacter asiaticus (*C*Las) and Americanus (*C*Lam) causal agents of the Huanglongbing (HLB), the most devastating disease of citrus worldwide [15,16]. Curiously, the spread of *D. citri* and the HLB pathogen in the two biggest citrus-producing states in the United States (Florida and California) has been unexpectedly different. In Florida, ACP were first detected in 1998, with HLB following shortly after in 2005 and quickly spreading throughout the major citrus production areas of the state. In California, ACP were introduced in 2008, and HLB followed in 2012 in residential citrus trees [17]. Currently, in California, HLB has not yet been detected in commercial citrus. The variation in establishment of the pathogen in citrus between the regions could be caused by multiple factors, but differences in climatic conditions are likely a key factor. 

*D. citri* are capable of both long- and short-distance dispersal. Long-distance dispersion (up to 2.4 km) by *D. citri* was demonstrated in previous flight mill studies [18], observational studies [19], and marking and recapture field studies [20]. Additionally, previous works have suggested that long-distance dispersion can be assisted by wind currents [5,21]. It has also been documented that *D. citri* commonly disperse over short distances (in the range of 5–300 m) [22,23,24,25,26,27]. Because HLB is a vector-transmitted disease, understanding the environmental factors that determine *D. citri* long- or short-distance dispersion is fundamental to understand HLB spread. Primary HLB spread (between citrus orchards) is caused by the long-distance dispersion of infected *D. citri*, while secondary spread (plant to plant movement or within orchards) is mediated by short-distance movement of infected, colonizing *D. citri* populations [27,28,29]. However, despite its importance, the effects of environmental factors that determine if a psyllid engages in long- or short-distance dispersion or that may influence flight capacity has not been thoroughly addressed through the wide variety of climatic conditions were this insect is distributed. 

Temperature and humidity influence *D. citri* flight activity [5,23,26,30,31,32]. However, the effect of humidity is still not clear. To date, one study has shown a negative correlation between humidity and propensity for take-off [26], while another showed no effect of humidity on the same parameter [30]. Likewise, most field study results suggest that *D. citri* dispersion is negatively correlated with relative humidity [23,31,32], but one study suggested a positive correlation [33]. Furthermore, it is still unknown if humidity alone directly affects *D. citri* flight capacity. Temperature, however, is known to play a key role in the dispersion of *D. citri*. Temperature is associated with *D. citri* short-distance dispersion and flight initiation [26,30]. Previous flight mill experiments conducted on *D. citri* under temperatures <30 °C showed an increase in flight activity up to 28 °C [5]. However, the previous flight mill studies only accounted for a limited range of temperatures (15–28 °C) and a high relative humidity, simulating Florida’s conditions and therefore did not account for California and Arizona conditions where high temperatures and low humidity are common. *D. citri* is able to survive at high temperatures and low humilities, suggesting a lower net water loss rate than other insects with similar sizes [34]. Nevertheless, the effect of high temperatures (>30 °C) or extremely high temperatures (>40 °C) under low relative humidity (RH) on the flight capacity of *D. citri* is still unknown. Thus, the objective of this study was to quantify the flight capability of *D. citri* in flight mills under different temperatures and RH levels similar to the maximum temperatures experienced in the hot summers of Southern California and other regions where this is applicable. To study this, we created a novel, temperature-controlled flight mill device. 

## 2. Materials and Methods

### 2.1. Insects

The insects used in this study were obtained from the insectary and quarantine facility at UC Riverside. The colony was established in 2012 from insects collected on untreated curry plants at a private residence in Azusa, California. Since then, the colony has been maintained on curry (*Bergera koenigii* L.) and Mexican lime (*Citrus aurantifolia*) plants without exposure to insecticides. The colony was maintained in controlled conditions at 26–28 °C, 30–40% RH, and a photoperiod of 14:10 (L:D). To ensure that the colony was free of *C*Las, the colony was periodically tested by real time PCR to confirm the absence of *C*Las. For use in flight mills, young adults (4–14 day old) were selected for the assays according to the procedure described by [18]. Only blue–green morphs were used because they have been previously shown to be the most capable of long flights [18,35].

### 2.2. Flight Mill

**Figure 1:**
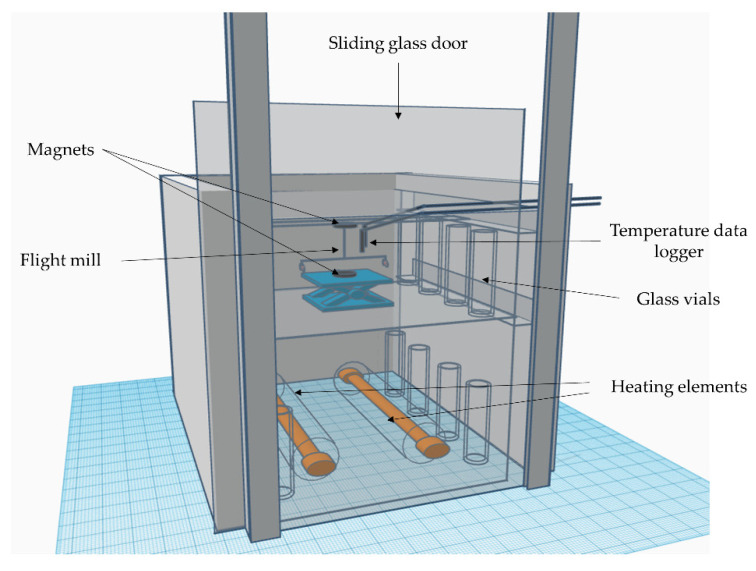
Diagram of the temperature-controlled flight mill used to test the flight capacity of *Diaphorina citri* under different temperatures.

A temperature-controlled flight mill was constructed based on the flight mill apparatus described by Martini et al. 2016 [18]. Briefly, this apparatus consisted of an optic fiber horizontal axis (10 cm) fixed through the eye of a sewing needle and suspended on a piece of glass with a magnet. To securely suspend the sewing needle and avoid the fiber from flipping horizontally, an additional magnet was positioned 1 cm below. Magnets allow for the rotation of the sewing needle with minimum friction [2]. The flight mill was enclosed in a glass chamber constructed from 30.48 × 40.64 cm transparent, tempered glass sheets (Contractors Wardrobe, Valencia, CA, USA) (Figure 1). The glass sheets were joined together with instant epoxy (JB Weld, Sulphur, TX, USA), and 1.9 cm aluminum framing angles (Everbilt/Home Depot, Atlanta, GA, USA) were used to brace the joints of the box construction. Overlapping framing angles allowed for one glass sheet to be used as a door to the chamber by sliding the sheet upwards. The bottom of the chamber was not glued to the glass sheet below, which allowed for air circulation. In order to control the temperature, a heat source and a thermostat controller were integrated into the glass chamber using the heating elements of a small oven (KitchenSmith, Quebec, QC, Canada), a digital proportional-integral-derivative temperature controller, and a solid-state relay (InkBird ITC-100 PID Temperature Controller, Shenzhen, China). To further control the maximum heat output, a pulse width modulation controller (RioRand) was used (Figure 1). To prevent excess heat loss when opening the door, an acrylic sheet with a 10.16 × 10.16 cm cutout was affixed to the outside of the sliding glass door. Temperature (Lascar data logger, Erie, PA, USA) and humidity (Ibutton, Australia) within the chamber were recorded for all flights. To assist in maintaining a stable temperature within the chamber, the heating elements were put inside borosilicate glass tubes (SunWo, Guangdong, China), and a sheet of glass was secured below the flight mill. The outside of the chamber was surrounded with three sheets of rigid foam insulation (Carlisle Construction Material, Carlisle, UK), and 18 glass vials were placed inside the chamber to minimize the heat loss by retaining heat [27].

All flight mill assays were performed in a temperature-controlled, windowless room designed to maintain insect colonies and eliminate the influence of natural diurnal light. The illumination of the room was provided by linear fluorescent 500 W lights (F32T8/TL950/ALTO Philips, Amsterdam, The Netherlands). The average light intensity in the flight mill was 0.23 µmol m^−2^ s^−1^. ACP adults were held before assays on curry plants (*Bergera koenigii* L.) and gradually acclimated to the target temperature over 1.5 h before starting the experiment inside an incubator (Heratherm^TM^, ThermoScientic, Langenselbold, Germany). Once acclimated, psyllids were immobilized using an ice pack covered with Kimwipes (Kimberly-Clark Professional, Roswell, GA, USA). Then, under a microscope, an optical fiber was attached to the pronotum using a small amount of nontoxic, washable glue (Elmer’s products, Columbus, OH, USA). The optic fiber was then attached to the end of the horizontal axis fiber of the central flight mill wand so that the psyllid faced 90 degrees from the horizontal axis. A dead psyllid was attached to the other end of the horizontal axis to act as a counterbalance.

All psyllids were allowed 10 min to fly after attachment and were considered “non-flyers” after 5 min of no flight activity. Psyllids were removed and the assay was terminated when the insect able to fly ceased flight for 5 min. The duration of the flights was recorded with a stopwatch, and the number of rotations was visually recorded for each flight for each insect. Psyllids were sexed at the end of the assay. The distance flown was calculated by multiplying the number of rotations by 10π. Temperatures were chosen in accordance to conditions observed in the summer of Southern California. Temperatures evaluated were (26, 32, 37, 40, 43, and 46 °C). Each temperature was tested at two relative humidity levels: low (30 ± 10%) and high (83 ± 7%). Low humidity consisted of the ambient RH in the laboratory at UC riverside. High humidity was obtained by inserting one extreme of a hose in the flight mill chamber with the other extreme connected to a mist humidifier (MI-AH001, Shenzhen Nearby Express technology Development Co., Ltd., Shenzhen, China). The flight mills were performed between 9:00 and 14.00 hours from 13 April 2020 to 24 July 2020 for low RH and from 11 December 2020 to 16 February 2021 for high RH.

### 2.3. Statistical Analysis

Due to considerable variability among the flight duration for individual psyllids, we divided the tested insects in three different categories according to previous works [18,35]: the categories used were: (1) non-flyers, (2) short flyers (psyllids that flew ˂60 s), and (3) long flyers (psyllids that flew ≥60 s). To compare if the proportion of psyllids from the three categories varied between temperatures, we used a Pearson´s chi squared test. Subsequently, for categories of short flyers and long flyers, we ran general linear models GLM followed by DMS tests in order to identify differences in flight duration, distance flown, and flight speed using temperature, RH, and psyllid sex as fixed variables. However, psyllid sex was later removed to simplify the models because this factor did not show significant effects in any of the tested variables. Finally, we assessed the relation between temperature and distance flown by using a linear regression for short and long flyers at low and high humidity. Data were analyzed using the SPSS v.24 statistical software package (IBM Corp 2016, Armonk, NY, USA). Data for flight duration and distance flown were transformed by log10 to decrease heteroscedasticity and achieve normality. Speed for short flyers was transformed with √x + 0.5.

## 3. Results

ACP were able to fly at all tested temperatures. The proportion of non-flyers did not differ between temperatures, but the proportion of short flyers and long flyers was significantly different between the different tested temperatures for low RH (χ^2^ = 45.0, df = 5, and *p* ˂ 0.001) and high RH (χ^2^ = 21.249, df = 5, and *p* ˂ 0.001) (Table 1). The highest frequency of short flyers was observed at 46 °C at low and high RH. For long flyers, the highest frequency was observed at 40 °C in low RH and 37 °C in high RH. The lowest frequency of long flyers was at 46 °C, where no long flights were observed at low or high RH (Table 1).

Pairwise comparisons between low vs high humidity per temperature only showed significant differences for 26 °C (χ^2^ =7.05, df = 1, and *p* = 0.008) and 40 °C (χ^2^ = 2.625, df = 1, and *p* = 0.001) where low humidity favored the propensity to engage in long flights (Supplementary Table S1).

For short flyers, flight duration was significantly affected by temperature (F = 3.463, df = 5, and *p* = 0.005) but not by RH (F = 3.369, df = 1, and *p* = 0.068), and there were no interactions between temperature and RH (F = 1.115, df = 5, and *p* = 0.322) Flight duration was longer for short flyers at 43 °C compared to other temperatures at low humidity (Table 2). Similarly, distance flown was longer for short flyers at 43 °C when compared to the other tested temperatures (F = 2.313, df = 5, and *p* = 0.044) (Table 2). Distance flown was not affected by RH (F = 0.661, df =1, and *p* = 0.417), and there were no interactions between temperature and RH (F = 1.438 df = 5, and *p* = 0.211). Moreover, for short flyers, the model showed that flight speed was affected by temperature (F = 6.448, df = 5, and *p* = 0.000), RH (F = 19.319, df = 1, and *p* = 0.000) and the interaction between temperature and RH (F = 5.997, df = 1, and *p* = 0.000). ACP were slower at 46 °C when compared to all tested temperatures. Additionally, ACP at high RH were slower than insects at low RH (Table 2).

For long flyers, flight duration was significantly affected by temperature (F = 7.024, df = 4, and *p* ≤ 0.001) but not by RH (F = 0.104, df = 1, and *p* = 0.748) or temperature × RH (F = 0.986, df = 1, and *p* = 0.418). Flight duration was significantly higher at 26 °C when compared to the other tested temperatures, except for 32 °C. There were no significant differences between 32, 37, and 40 °C, but flight duration at 43 °C was significantly lower when compared to all temperatures (Table 2). Likewise, the distance flown for long flyers was significantly affected by temperature (F = 6.494, df = 4, and *p* ≤ 0.001) but not by RH (F = 3.083, df = 1, and *p* = 0.082) or temperature × RH (F = 1.125, df = 1, and *p* = 0.348). ACP at 26 °C flew significantly longer than insects at 40 and 43 °C but not than ACP at 32 and 37 °C. Distance flown was significantly lower at 43 °C when compared to the other tested temperatures (Table 2). The maximum values recorded for distance flown were both performed by females at 26 °C with 2188 m at low RH and 2598 m at high RH. (Table 2). Flight speed was not different between temperature treatments for long flyers (F = 1.151, df = 4, and *p* = 0.339). However, long flyers at high RH were faster than insects at low RH (F = 24.718, df = 1, and *p* ≤ 0.001) (Table 2). 

**Figure 2:**
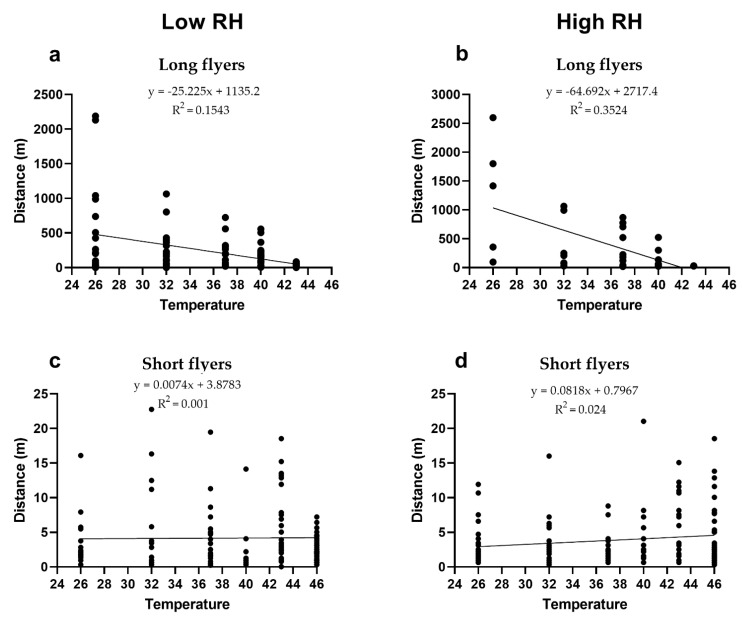
Linear regression showing the effect of temperature (°C) on distance flown by *D. citri* for: (**a**) long flyers at low RH, (**b**) long flyers at high RH, (**c**) short flyers at low RH, and (**d**) short flyers at high RH.

A linear regression analysis showed that there was a negative correlation between temperature and distance flown for long flyers at low and high RH (Figure 2a,b). However, there was no correlation between temperature and distance flown for short flyers at low or high RH (Figure 2c,d). 

## 4. Discussion

Understanding how temperature and humidity affects *D. citri* flight capacity is fundamental to understand and predict its dispersion under different environmental conditions. To our knowledge, this is the first study to quantify the flight capacity of *D. citri* under high temperatures (>40 °C) at a relative humidity similar to that experienced in the semi-arid climates of regions within citrus-producing regions of California and Arizona, where the spread of Huanglongbing is an imminent threat. 

Identifying the maximum threshold temperature at which flight capacity of *D. citri* decreases is of critical importance to understand its dispersion in zones experiencing high temperatures. Our data were in accordance with previous works conducted with flight mills on *D. citri* [5,18] below 30 °C, with a majority of short flyers and average flight distance covered for long flyers comprised between 50 and 320 m. Our data give a more complete picture of *D. citri* flight patterns as a function of temperature: flight initiation starts at 18 °C, flight capacity increases up to 28 °C [5], and then flight capacity starts to decline after 32 °C. Finally, at temperatures above >43 °C, insects did not engage in long-distance flights. Thus, as evidenced by the presented data, *D. citri* has the capacity to perform long-distance flights in hot weather as long as temperatures do not surpass 43 °C, regardless of the relative humidity. Interestingly, the linear regression analysis showed that for those psyllids able to engage in long-distance flights, distance dispersed decreased as temperature conditions increased. 

The most favorable temperature for long-distance flight was 26 °C, in which maximum covered distance was 2598 m. Then, from 32 to 40 °C, the maximum distance flown was in the range of 1064–557 m and finally showed a marked decrease at 43 °C, where the maximum observed distance was only 87 m. According to these results, even though *D. citri* is able to perform long flights at high temperatures, dispersion decreases when temperatures increase to above 40 °C. Furthermore, in addition to the negative effect of temperature in *D. citri* dispersion, high temperatures can reduce *C*Las titer and *C*Las acquisition by *D. citri*, as well as having a detrimental effect in *D. citri* populations [36,37,38,39,40,41,42,43]. Accordingly, the rapid spread of HLB in regions such as Florida in USA or the São Paulo state in Brazil could be partially explained by the absence of extreme high temperatures. 

Temperature affects seasonal flight activity and diurnal flight patterns [26,44]. Flight activity of *D. citri* is more pronounced in the afternoon hours, when temperature and light intensity values are their highest points for the day [26,44]. However, this information has been obtained in temperatures below 32 °C. Our results suggest that extreme high temperatures may also influence *D. citri* diurnal flight patterns, decreasing the efficiency of dispersion in very hot days. Further experiments in field conditions are needed to support this hypothesis because this information may impact crop management techniques such as spray timing. 

High temperatures (>40 °C) also affect insect physiological processes [45]. As such, the observation of temperatures above 40 °C significantly decreasing *D. citri* flight capacity was not unexpected. Previous studies have showed high temperatures as detrimental for different *D. citri* life traits such as survivorship, oviposition, longevity or hatching [41,42,46,47]. Exposure to temperatures above 40 °C induces the transcription of heat shock proteins and may cause ATPase and protein denaturation [40,41] which are critical for survival. Consequently, *D. citri* can tolerate short periods above 40 °C, but constant exposure to extreme temperatures (>40 °C) has been shown to be lethal [41,47]. Even for small insects such as psyllids, a rise in temperature favors water loss and desiccation. Thus, the effect of temperature on flight capacity is likely to be caused by the combined effects of heat and water stress. 

Although the exact contribution of water stress to the decrease in flight capacity observed in this study is unknown, we suspect the effect of water stress to be low for two reasons. First, *D. citri* is capable of tolerating high temperature and low humidity levels and its survival under these conditions is higher than the reported for insects with similar size [34]. Second, if a high net water loss was the case for *D. citri,* insects at high temperatures and high humidity would have performed better than insects at high temperatures and low humidity. However, this was not the case, as insects flew similar distances in low and high relative humidity.

In the presented data, there was considerable variability in the propensity to engage in long or short flights among individuals. However, the distribution for the number of flight attempts was negatively skewed, with a smaller proportion of attempts for long-distance flights. This suggests that short-distance movement is preferred over long-distance dispersion. This is in agreement with previous studies that have described frequent short-distance movements of *D. citri* in field conditions [22,23,24,25,26,27]. It is critical to understand what factors motivate *D. citri* to engage in short or long flights. The results of this work suggest that temperature influences the propensity to engage in long-distance flights, as well as the duration of those flights. Moreover, our data suggest that low relative humidity increases the propensity of *D. citri* to engage in long-distance flights—but only at 26 and 40 °C. An increased propensity to engage in long flights at low humidity at 26 °C, when added to the high flight capacity observed, suggests that this temperature and humidity combination is optimal for *D. citri* dispersion. This is in agreement with recent data showing a higher number of migrating psyllids during spring, when low humidity and cool temperatures were present in citrus groves in field conditions in Brazil [32]. In addition, other factors such as barometric pressure may also influence flight behavior, with a drop in pressure associated with dispersal and flight activity [5,30]. Since primary spread is caused by the long-distance dispersion of HLB-infected *D. citri* [28,29], increased long-distance migration under low humidity, cool temperature, and drops in barometric pressure may also favor HLB spread. Identifying these risk conditions according to the citrus-producing area is essential and could improve decision making for HLB and *D. citri* control. 

Finally, to fully understand the dispersion of *D. citri* under different temperatures in field conditions in addition to flight capacity, other components of flight behavior that cannot be evaluated with a flight mill need to be considered. For example, how high temperatures affect the take-off propensity or how wind or other stimuli could influence flight orientation are questions that need to be addressed in further studies.

## 5. Conclusions

Our study provides valuable information describing the capacity of *D. citri* to fly in different environmental conditions. Combined with other abiotic data, this information can be used to improve input data in simulation models used to forecast *D. citri* and HLB dispersion, as well as for risk evaluation. Furthermore, the information provided here could lay the groundwork for optimizing management surveillance protocols by considering the particular conditions where this insect vector is currently found.

The obtained results indicated that temperature affects the flight capacity of *D. citri*. High temperatures similar to those experienced in citrus-producing regions throughout Southern California and Arizona would be detrimental for *D. citri* flight capacity.

## Figures and Tables

**Table 1:** Percentage of *D. citri* that did not engage in flight (non-flyers), engaged in flight for less than 60 s (short flyers), or engaged flights for more than 60 s (long flyers) when tested in a temperature-controlled flight mill under low or high relative humidity. Means followed by different letters within the same column are significantly different within each humidity category (Duncan χ^2^ test *p* ˂ 0.05).

Temperature	Non-Flyers	Short Flyers	Long Flyers	N
	%	%	%	
Low RH				
26	11.12 a	48.8 b	40.0 b	45
32	10.6 a	50.0 b	39.4 b	38
37	5.3 a	47.3 b	47.3 b	38
40	8.2 a	21.6 c	70.2 a	37
43	11.7 a	60.4 b	27.9 b	43
46	12.5 a	87.5 a	0 c	40
High RH				
26	17.9 a	69.2 b	12.8 a	39
32	17.1 a	62.8 b	20.0 a	35
37	15.6 a	50.0 b	34.3 a	32
40	20.0 a	51.4 b	28.5 a	35
43	14.7 a	73.5 b	11.7 a	34
46	7.8 a	92.1 a	0 b	38

**Table 2:** Flight capacity parameters for *D. citri* engaged in flight for less than 60 s (short flyers) or engaged flights for more than 60 s (long flyers) when tested under six different temperatures and low and high relative humidity. Means in the same column followed by different letters are significantly different by DMS test *p* ˂ 0.05. Comparisons were separately performed for low and high relative humidity.

Temperature	Short Flyers			Long Flyers			
	Flight Duration (min)	Distance Flown (m)	Flight Speed (km/h)	Flight Duration (min)	Distance Flown (m)		Flight Speed (km/h)
Low RH	Mean	Mean	Mean	Mean	Mean	Maximum	Mean
26	0.14 b	3.06 b	1.36 a	37.91 a	500.81 a	2188	0.72 b
32	0.19 b	5.00 b	1.65 a	18.97 ab	281.45 ab	1064	0.99 b
37	0.18 b	4.70 b	1.56 a	13.05 b	191.36 ab	723	0.97 b
40	0.18 b	2.98 b	1.36 a	10.39 bc	152.18 b	557	0.92 b
43	0.34 a	7.54 a	1.40 a	2.49 c	32.71 c	85	0.77 b
46	0.19 b	2.59 b	0.90 b	0 *	0 *	0 *	0 *
High RH							
26	0.17 b	3.02 b	1.14 b	65.00 a	1253.99 a	2598	1.15 a
32	0.18 b	3.19 b	1.04 b	22.08 ab	385.59 ab	1064	1.18 a
37	0.14 b	2.85 b	1.24 b	17.50 b	339.26 ab	869	1.21 a
40	0.23 ab	4.22 b	1.07 b	7.66 bc	147.95 b	302	1.17 a
43	0.26 ab	4.77 b	1.07 b	1.59 c	32.18 c	37	1.24 a
46	0.25 ab	4.50 b	1.11 b	0 *	0 *	0 *	0 *

* These values were not included in the analysis because long flights were not observed at 46 °C.

## Data Availability

Data are presented within the article and raw data is available upon request.

## Supplementary Materials

The following are available online at https://www.mdpi.com/article/10.3390/insects12050394/s1, Table S1: Pairwise comparison of *D. citri* short and long flights.
