# Supplementary material for: High Temperatures Decrease the Flight Capacity of Diaphorina citri Kuwayama (Hemiptera: Liviidae)
Source: Insects. 2021 Apr 29;12(5):394. doi: 10.3390/insects12050394 (PMC8145625; doi:10.3390/insects12050394)
Supplement: Supplementary file 1 [file insects-12-00394-s001.zip › Table S1.pdf]

Supplementary table 1. Pairwise comparisons for the proportion of *Diaphorina citri* that engaged in flight for less than 60s (short flyers), or engaged flights for more than 60s (long flyers) when tested in a temperature-controlled flight mill under low or high relative humidity. Means followed by different letters within the same column are significantly different (χ^2 test $P<0.05$).

**Temperature**	**Low RH**		**High RH**		**P**
	Short flyers %	Long flyers %	Short flyers %	Long flyers %	
26	55	45	84	16	0.00
32	56	44	76	24	0.09
37	50	50	59	41	0.46
40	24	76	64	36	0.00
43	68	32	86	14	0.10
46	100	0	100	0	*

* This comparison was not performed because there were no long flights at 46 °C
